# Surgical Management of Giant Intracranial Meningiomas

**DOI:** 10.5152/eurasianjmed.2021.20155

**Published:** 2021-06

**Authors:** Soner Yaşar, Alparslan Kırık

**Affiliations:** Department of Neurosurgery, University of Health Sciences, Gülhane Education and Research Hospital, Ankara, Turkey

**Keywords:** Meningioma, Giant, Surgery, Brain

## Abstract

**Objective:**

Giant intracranial meningiomas are a challenge for neurosurgeons because of their size and location in the cranium. Difficult tumor dissection and encasement of important neurovascular structures make them a horrible nightmare. The aims of this study are to present our giant intracranial meningioma series and to compare our experience using advanced surgical technology with the current literature.

**Materials and Methods:**

The data of patients with the diagnosis of giant intracranial meningioma between 2014 and 2020 who underwent surgical treatment were retrospectively reviewed. The demographic, radiological, and surgical characteristics of patients were documented. The size and location of tumors as well as surgical technique were analyzed in detail.

**Results:**

A total of 61 patients with intracranial meningioma underwent surgical treatment over a 7-year period, and 10 (16.4%) tumors were larger than 5 cm in diameter, which were classified as giant meningioma. Seven patients were male and 3 were female, with a mean age of 64.9 years. Four tumors were located at the skull base. Histological diagnosis was meningioma World Health Organization grade I in 7 patients and grade II in 3 patients. Simpson grade 1 resection was achieved in 6 patients and grade 2 resection in 4 patients. No mortality was observed.

**Conclusion:**

Careful surgical planning should be made for giant intracranial meningiomas. Their location, adjacent neurovascular structures, and vascular supply affect the resection level of these giant tumors. Simpson grade 1 resection is seldom possible for skull base meningiomas.

## Introduction

Meningiomas are the most common intracranial benign tumors, arising from the arachnoid cap cells. They are relatively slow-growing extra-axial tumors.^1^,^2^ The most common locations for these tumors are convexity, parasagittal/falcine area, sphenoid wing, tuberculum sellae, and posterior fossa. These tumors have clinical symptoms related to their location within the cranium or increased intracranial pressure. Because they are slow growing, radiological diagnosis is usually made when the tumor reaches a large volume.^1^–^3^

Computed tomography (CT) and magnetic resonance imaging (MRI) are the main radiological tools for the diagnosis of an intracranial meningioma. MRI clearly defines the origin and borders of the tumor.^4^ It is also used to plan the surgical approach for removal of the tumor. CT reveals the bone involvement of meningiomas, such as erosion or hyperostosis. Giant meningiomas may also be seen in plain skull X-rays when they are calcified or cause bone changes.^5^,^6^ Angiography is useful to detect the vascular supply of the tumor and to obliterate feeding vessels before surgery, which may help to achieve total resection of the tumor.^4^–^6^

Meningiomas may be large (>3 cm) or giant (>5 cm) at the time of diagnosis.^7^,^8^ Giant meningiomas are different than the others because of their huge size, causing increased intracranial pressure, and close proximity to critical anatomical structures.^8^ Therefore, they are always a challenge for neurosurgeons.

Surgical removal is the main treatment method for giant intracranial meningiomas. The surgical approach for each tumor is tailored depending on its size and location for a safe and effective maximum tumor resection.^9^,^10^ Gross total resection is the main goal of surgical management. However, it is not always possible for giant meningiomas because of adherence and encasement of important neurovascular structures, such as the optic nerve, carotid artery, or superior sagittal sinus.^9^–^14^

The aims of this study are to present our cases with giant intracranial meningioma, to show the surgical challenges of these rare tumors, and to discuss our findings with the current literature.

## Materials and Methods

The data of patients who underwent surgical resection for intracranial tumor and diagnosed meningioma between 2014 and 2020 were retrospectively reviewed. Among them, patients who had an intracranial meningioma larger than 5 cm in maximum diameter were included in this study. The demographic, radiological, and surgical features of these patients were analyzed. Ethical approval was obtained from the Gülhane Scientific Ethics Committee for this study.

**Figure 1. a–d:**
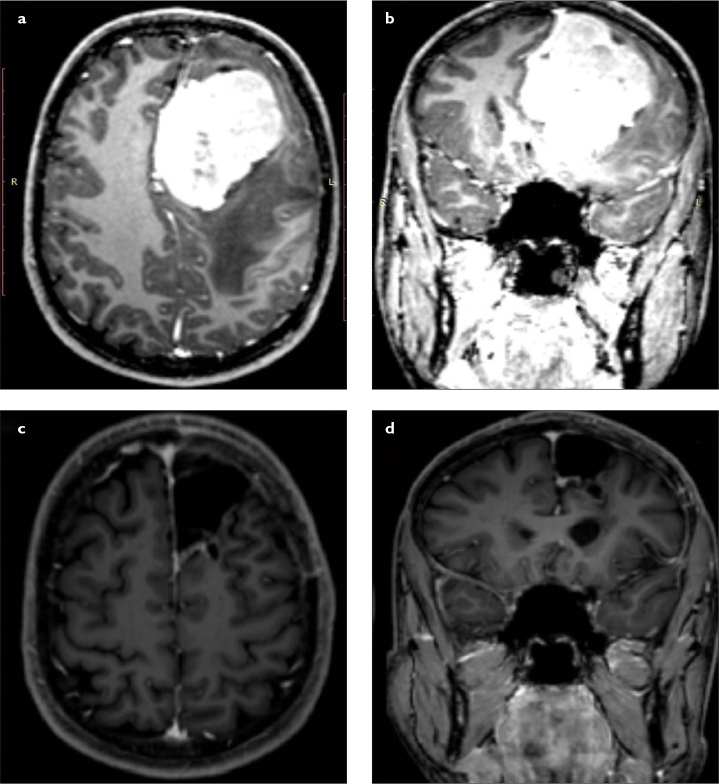
(a) Preoperative axial and (b) coronal MRI scans of a patient with giant left frontal meningioma originating from the falx cerebri. The patient underwent surgical resection using left frontal craniotomy. (c) Postoperative axial and (d) coronal MRI scans confirmed gross total resection. MRI, magnetic resonance imaging.

**Figure 2. a–d:**
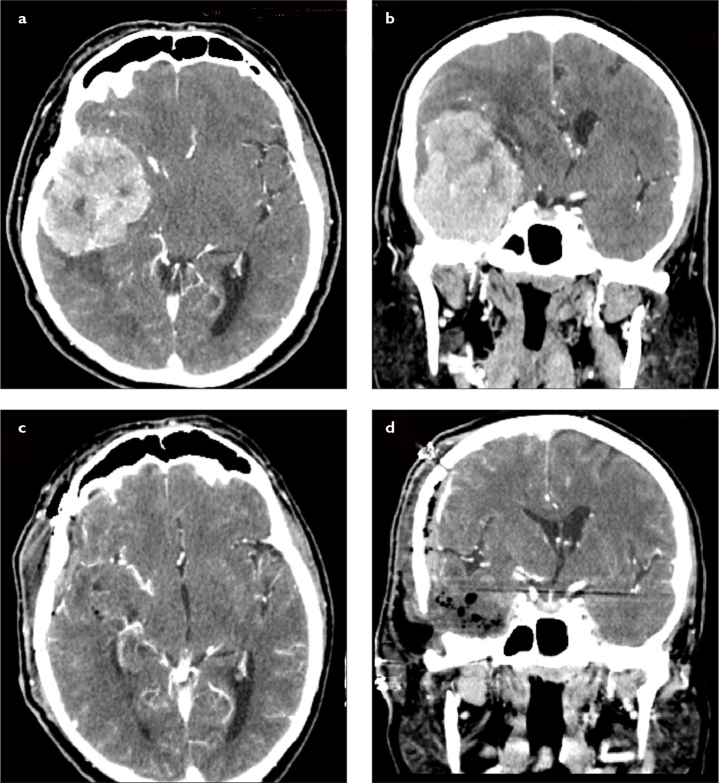
(a) Preoperative axial and (b) coronal CT scans (with contrast) of a patient with giant right temporal fossa meningioma originating from the skull base dura mater. There was a significant midline shift and brain edema. The patient underwent gross total resection using right temporal craniotomy. (c) Postoperative axial and (d) coronal CT scans (with contrast) confirmed gross total resection. The shift was improved and the resolution of brain edema was obvious. CT, computed tomography.

**Figure 3. a–d:**
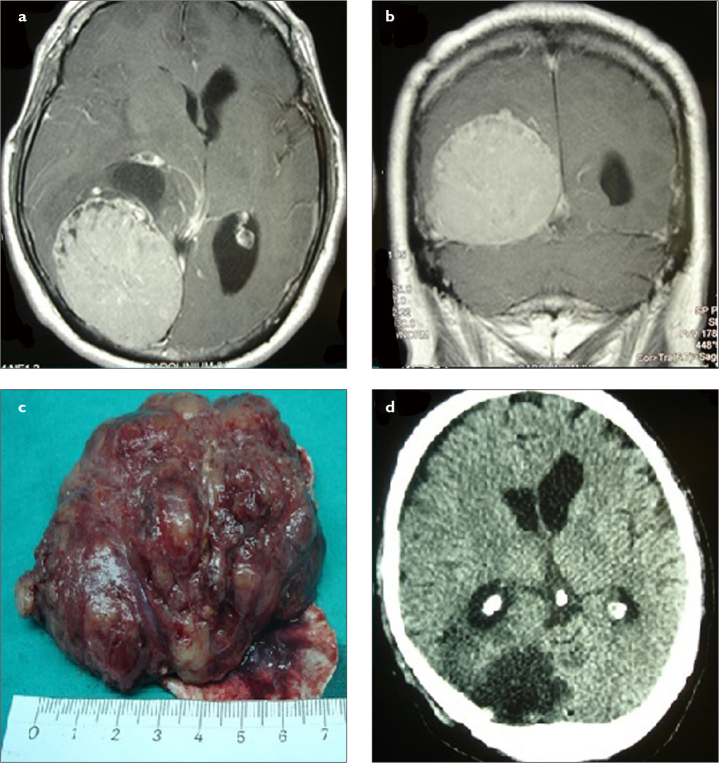
(a) Preoperative axial and (b) coronal MRI scans of a patient with giant left posterior parietal meningioma. There was a significant compression on the atrium of the left lateral ventricle. The patient underwent gross total resection including dura mater using left parietal craniotomy. (c) The tumor was larger than 6 cm. (d) Postoperative axial CT scan confirmed the total resection of tumor. CT, computed tomography; MRI, magnetic resonance imaging.

Clinical presentation, initial neurological examination, and preoperative CT and MRI of patients were recorded (Figures 1–3). Craniotomy was planned for each patient before surgery, and a neuronavigation system was set up at the beginning of operation. The neuronavigation system was used in all procedures. Microsurgical techniques were used to reach and remove the tumor (Figure 3). Endoscopic technique was used only in one case with a giant skull base meningioma in which we could not achieve Simpson grade 1 resection after classical microscopic technique. Simpson scale was used to describe the extent of resection.

## Results

A total of 61 patients were identified with the diagnosis of meningioma over a 7-year period. Among them, 10 (16.4%) patients had a diagnosis of giant intracranial meningioma. Seven patients were male and 3 were female, and the mean age was 64.9 years (range, 30–83 years). The size and location of tumors are shown in Table 1.

All patients underwent surgical removal of the tumor. A total of 11 surgical procedures were performed in 10 patients. Tumors were removed in 1 session in 9 patients and 2 sessions in 1 patient. The tumor destructed the cranial bony structure and reached to subcutaneous area in 1 patient. The tumor was located at the skull base in 4 patients, in the anterior fossa in 2 patients, on the sphenoid wing in 1 patient, and in the temporal fossa in 1 patient. Among them, Simpson grade 2 resection was achieved in 4 patients, whereas grade 1 resection was achieved in the 6 patients without skull base location. Preoperative feeding artery embolization was only performed in 1 patient, and successful surgical resection was performed in this patient without massive bleeding.

No patient died after surgery and no neurological deterioration was observed in any patient after surgery. Cerebrospinal fluid (CSF) collection was seen at the surgical site in 2 patients and treated conservatively.

## Discussion

We presented the clinical outcomes of 10 patients who underwent surgical management of giant intracranial meningioma. We focused on surgical challenges of these rare tumors and emphasized the knowledge of relevant anatomy and surgical techniques.

Meningiomas are extra-axial tumors that usually present as a solitary tumor but may also be associated with other tumors, such as gliomas or schwannomas.^15^,^16^ They are mostly seen in adults but seldom diagnosed in pediatric patients.^17^ The differential diagnosis of meningiomas are solitary fibrous tumors, schwannomas (in the posterior fossa), gliomas, hemangiopericytomas, dural metastases, chordomas, and pituitary macroadenomas (in the skull base).^18^

There is no exact definition of giant meningioma in the literature. Some authors defined giant meningioma as a tumor >4.5 cm; others defined it as larger than 5 cm, 6 cm, or 7 cm in maximum diameter.^7^–^13^ Some authors named these tumors huge meningiomas^12^, but in the majority of publications, tumors >5 cm are usually identified as giant meningiomas. Therefore, we accepted 5 cm as the lower limit of diameter in giant meningioma cases.

Most meningiomas are slow-growing tumors with a growth rate of approximately 2.4 mm per year.^14^,^19^ Because of their slow growth, they seldom cause clinical symptoms until the tumor becomes large in size, especially when they are located in silent brain areas.

Giant meningiomas are extremely rare and difficult to resect totally. These tumors are considered complex lesions because of their compressive effects on the brain tissue, increased intracranial pressure, and hemodynamic changes secondary to vascular compression. A variable amount of vasogenic edema is shown in adjacent brain tissue in more than half of meningioma cases.^20^ Preoperative corticosteroid treatment may reduce tumor water content and peritumoral edema.^21^ There are 3 important issues related to giant meningioma surgery: 1) longer surgical times associated with increased blood loss, 2) difficulty in releasing encased or involved main cerebral arteries and cranial nerves, and 3) difficulty in obtaining better tumor exposure because of bridging veins and surrounding arteries.^22^,^23^ Therefore, preoperative precise information about the location of important arteries and nerves is crucial for a safe surgery of giant intracranial tumors. Surgery of a giant meningioma should always begin with the coagulation of feeding vessels. Another tricky point of surgery is to avoid any damage on the tensed brain tissue during approach, dissection, and tumor removal stages. Microsurgical techniques should be applied during these stages. The origin of the meningioma should always be the first target of surgery if possible. Moreover, tiny incisions in the dura mater or opening the arachnoid cisterns after craniotomy may allow gradual CSF drainage and decrease intracranial pressure. After the dural opening, small cottonoids may be placed around and beneath the meningioma under microscope, and the brain-tumor interface is protected by these cottonoids until the release of tumor tissue from the brain. Gradual separation of adhesions between the brain and the tumor reduces brain traction during tumor resection. A cavitron ultrasonic aspirator may be used to debulk the tumor.^19^ Following enough reduction of tumor volume, the brain tissue pushes the tumor mass more and more toward the neurosurgeon and facilitates the removal of tumor. Involved dura and dural rim should also be removed to achieve Simpson grade 1 resection. For giant skull base meningiomas, CSF drainage from the basal arachnoid cisterns using transsylvian dissection allows excellent brain relaxation. Tumor removal may be made from the inside out. Endoscopic techniques in skull base meningiomas provide early tumor devascularization and detachment of tumor from the skull base.^24^

Selection of surgical approach for giant intracranial meningiomas mainly depends on the location of the tumor. Giant convexity meningiomas require large enough craniotomy for safe tumor dissection and removal.^25^ The size of craniotomy must be greater than the tumor diameter. This craniotomy should be tailored based on the preoperative CT and MRI scans of the patient. The removal of giant skull base meningiomas should be planned based on the location of tumor and tumor size.^7^,^26^,^27^ Craniofacial approaches are appropriate for some skull base meningiomas.^28^ Giant anterior fossa or tuberculum sellae meningiomas may be resected using a pterional, subfrontal, or endoscopic endonasal approach. Ventricular meningiomas could be resected by endoscopic techniques using a tubular retractor (port) or by an interhemispheric transcallosal approach under microscopic view.^29^,^30^ In our series, we performed 11 surgical procedures in 10 patients. All patients underwent preoperative CT and MRI scans. Only 1 patient underwent preoperative embolization. Ten procedures were craniotomies with tumor removal using microsurgical techniques and 1 procedure was an extended endoscopic endonasal technique for a skull base meningioma that was subtotally removed with classical microsurgical techniques.

A few series of giant intracranial meningioma were reported previously.^9^,^12^,^13^ Others were presented as case reports.^6^,^22^,^23^,^25^,^26^,^31^,^32^ Tuna et al.^12^ reported 93 patients with huge intracranial meningiomas, and they classified meningiomas >6 cm in minimum diameter as huge meningiomas. Mean age was 48.7 years, and mortality was 3.2%. They concluded that the huge size of meningioma negatively affects the extent of removal, recurrence rate, postoperative outcome, and mortality.^12^ Narayan et al.^9^ reported 80 patients with giant intracranial meningioma who were surgically treated over a 20-year period. They considered tumors >5 cm in maximum dimension as giant intracranial meningioma, similar to our study. The mean age was 56 years in this study, and the mortality rate was 5%. They concluded that young age, male sex, use of neuronavigation, and skull base location positively influence recurrence-free survival, whereas Simpson grade 3 or grade 4 excision and poor histological grade negatively influence recurrence-free survival.^9^ Özsoy et al.^13^ reported their series of 56 patients with giant meningiomas over a 15-year period and emphasized that the size of tumor and the amount of surgical excision affect the rate of recurrence, mortality, and morbidity. The mean age was 49.75 years, and the mortality was 7.1%.^13^ Although our series is smaller and older than Özsoy et al.^13^ and Narayan et al.^9^, we achieved Simpson grade 1 or 2 resection in all cases, and we had no mortality after surgeries. Attia et al.^33^ reported their experience with 22 giant anterior clinoidal meningiomas, and they defined these tumors as globular tumors with a maximum diameter of 5 cm or larger, centered around the anterior clinoidal process. They performed a skull base approach with extradural unroofing of the optic canal, extradural clinoidectomy, transdural tumor debulking, early optic nerve decompression, and early identification and control of important neurovascular structures. They achieved total tumor resection (Simpson grade 1 or 2) in 30.4% of surgeries without mortality.^33^ In our series, we had only 1 patient with a giant meningioma arising from the anterior clinoid process, and that patient underwent Simpson grade 2 tumor resection using microsurgical techniques. Behari et al.^8^ reported their experience with large/giant meningiomas of the posterior third ventricular region in 4 patients and suggested an occipital transtentorial approach for such tumors.^8^ A suboccipital transtentorial approach may be used for meningiomas located in the posterior mediobasal temporal region.^34^ If the tumor extends toward the intraorbital region, superior or lateral orbitotomies under navigation guidance are useful to safely remove the tumor.^35^ In our series, we had no patient with giant intraventricular or orbital meningioma.

The surgical treatment of giant intracranial meningiomas became satisfactory and safer with the use of advanced imaging techniques and intraoperative technologies, such as neuronavigation, 3-dimensional ultrasound, radiofrequency thermocoagulation, intraoperative angiography, and electrophysiological monitoring.^9^,^27^,^33^,^35^ These developments decreased the morbidity and mortality of these difficult cases. The extent of resection in meningiomas is always measured by Simpson grading in neurosurgery^9^, and we achieved gross total resection (Simpson grade 1 and 2) in all cases. A neuronavigation system provides the shortest way to reach the tumor and optimum craniotomy for safe resection. This also increases the resection level of the tumor and survival of the patient.

Complications are not frequent in giant intracranial meningioma surgery. Tumor bed hematoma, CSF collection or leak, and infection are the major complications of giant intracranial meningiomas.^9^,^11^–^13^ Insufficient coagulation of tumor bed may cause postoperative hematoma and neurological deficits. Inadequate dural closure may result in CSF collection or leak. In our series, we did not encounter hematoma or CSF fistula, but CSF collection occurred in 2 patients and was treated conservatively.

### Limitations

Low patient population is the major limitation of this study. Another limitation is the lack of a control group to compare the surgical results.

In conclusion, although giant intracranial meningiomas are rare, their surgical management is challenging and requires extreme caution for patient safety. Good anatomical knowledge and microsurgical skills are needed to overcome the risks of complications.

**Main Points**

- Giant meningiomas are very rare and challenging cases in neurosurgery.
- Good anatomical knowledge, extreme caution, and microsurgical skills are required for a safe and effective surgical treatment.
- Simpson grade 1 resection is seldom possible for skull base meningiomas.

## Figures and Tables

**Table 1:** Demographic, Radiological, Surgical, and Histological Characteristics of Patients who Underwent Surgery for Giant Intracranial Meningioma

Patient no.	Age (years)/sex	Location	Size (cm)	Origin	Resection grade (Simpson grade)	Histological diagnosis
1	30/Male	Left frontal	7 × 5.2 × 6	Falx cerebri	Grade 1	Meningioma WHO grade I
2	49/Male	Olfactory groove (skull base)	5.7 × 4.2 × 5	Anterior skull base dura	Grade 2	Meningioma WHO grade I
3	82/Male	Right posterior parietal	4.6 × 6 × 5.3	Convexity dura	Grade 1	Meningioma WHO grade II
4	78/Male	Anterior clinoid (skull base)	5 × 6 × 6	Skull base dura	Grade 2	Meningioma WHO grade I
5	83/Male	Right frontal	6.7 × 5.5 × 6.3	Convexity dura	Grade 1	Meningioma WHO grade I
6	53/Female	Left frontoparietal	6.4 × 7.8 × 5.6	Falx cerebri	Grade 1	Meningioma WHO grade I
7	70/Female	Left posterior parietal	7 × 5 × 7	Convexity dura	Grade 1	Meningioma WHO grade I
8	70/Female	Left temporal fossa	7.3 × 7.1 × 5.1	Sphenoid wing dura	Grade 2	Meningioma WHO grade I
9	72/Male	Left frontal	7.3 × 6.8	Convexity dura	Grade 1	Meningioma WHO grade II
10	62/Male	Right temporal fossa	6.5 × 5.5	Skull base dura	Grade 2	Meningioma WHO grade II

WHO, World Health Organization.
